# Preservation of Person-Centered Care Through Videoconferencing for Patient Follow-up During the COVID-19 Pandemic: Case Study of a Multidisciplinary Care Team

**DOI:** 10.2196/25220

**Published:** 2021-03-05

**Authors:** Line Silsand, Gro-Hilde Severinsen, Gro Berntsen

**Affiliations:** 1 Norwegian Centre for E-health Research Tromsø Norway; 2 Institute of Community Medicine UiT – The Arctic University of Norway Tromsø Norway

**Keywords:** person-centered care, rapid digitalization, health care, videoconferencing, persons with complex, long-term needs, COVID-19

## Abstract

**Background:**

The Patient-Centered Team (PACT) focuses on the transitional phase between hospital and primary care for older patients in Northern Norway with complex and long-term needs. PACT emphasizes a person-centered care approach whereby the sharing of power and the patient’s response to “What matters to you?” drive care decisions. However, during the COVID-19 pandemic, videoconferencing was the only option for assessing, planning, coordinating, and performing treatment and care.

**Objective:**

The aim of this study is to report the experience of the PACT multidisciplinary health care team in shifting rapidly from face-to-face care to using videoconferencing for clinical and collaborative services during the initial phase of the COVID-19 pandemic. This study explores how PACT managed to maintain person-centered care under these conditions.

**Methods:**

This case study takes a qualitative approach based on four semistructured focus group interviews carried out in May and June 2020 with 19 PACT members and leaders.

**Results:**

The case study illustrates that videoconferencing is a good solution for many persons with complex and long-term needs and generates new opportunities for interaction between patients and health care personnel. Persons with complex and long-term needs are a heterogeneous group, and for many patients with reduced cognitive capacity or hearing and vision impairment, the use of videoconferencing was challenging and required support from relatives or health care personnel. The study shows that using videoconferencing offered an opportunity to use health care personnel more efficiently, reduce travelling time for patients, and improve the information exchange between health care levels. This suggests that the integration of videoconferencing contributed to the preservation of the person-centered focus on care during the COVID-19 pandemic. There was an overall agreement in PACT that face-to-face care needed to be at the core of the person-centered care approach; the main use of videoconferencing was to support follow-up and coordination.

**Conclusions:**

The COVID-19 pandemic and the rapid adoption of digital care have generated a unique opportunity to continue developing a health service to both preserve and improve the person-centered care approach for persons with complex and long-term needs. This creates demand for overall agreements, including guidelines and procedures for how and when to use videoconferencing to supplement face-to-face treatment and care. Implementing videoconferencing in clinical practice generates a need for systematic training and familiarization with the equipment and technology as well as for an extensive support organization. Videoconferencing can then contribute to better preparing health care services for future scenarios.

## Introduction

The COVID-19 pandemic, which began in mid-March 2020, has created unprecedented challenges for society and health care systems worldwide. In Norway, as in many other countries, the government imposed extensive lockdowns and social distancing measures in public spaces to reduce the spread of the SARS-CoV-2 virus. Regarding health care services, most scheduled physical appointments and nonacute surgeries were cancelled to protect both health care personnel and patients. Nevertheless, it was necessary to accept new patients with acute or subacute conditions and follow up with long-term care patients. Hence, health care personnel were suddenly forced to rethink and find alternative solutions for face-to-face treatment and care of patients. This resulted in the swift implementation of telemedicine and, in particular, the increased use of videoconferencing.

Telemedicine is defined as communication over a distance in which video and audio are transmitted in near–real time. The provision of health care remotely by means of a video meeting is not new and has been well described; see for instance [1-7]. Despite this, previous research illustrates a paradox in telemedicine in terms of the low rate of adoption of the technologies despite strong policy-level strategies and many small-scale proof-of-concept examples [2,3,8]. In a study from 2017, Alami et al [3] found that the use of telemedicine in Norway from 2009 to 2015 did not exceed 0.5% of total outpatient activity at a regional level. The study identified three major themes affecting the implementation and use of telemedicine in Norway: (1) governance and strategy, (2) organizational and professional dimensions, and (3) economic and financial dimensions [3]. An example is the Norwegian Directorate of eHealth making changes to the reimbursement of videoconferencing in response to its increased and necessary use during the COVID-19 pandemic [9]. This is in accordance with previous studies that have shown that introducing video consultations is a complex change that disrupts long-established processes and routines [10,11].

Nevertheless, in recent years, telemedicine has attracted considerable interest as a means of delivering care to patients with long-term conditions [11,12]. The use of videoconferencing in the management of chronic diseases such as chronic obstructive pulmonary disease, diabetes, and depression has potential for improving health care services [1]. Almost all of this evidence pertains to selected samples of outpatients with chronic, stable conditions [10]. Consequently, the rapid spread of COVID-19 has accelerated the use of telemedicine for assessing, planning, coordinating, and performing treatment and care. The crisis seems to have lowered previous barriers to using telemedicine and to have led to the discovery of new ways of using digital health solutions in response to the crisis [12]. By using telemedicine, health care services can support not only secured care for patients with COVID-19 but also electronic prescriptions and triage of patients with COVID-19 in different phases. In addition, health care services can use telemedicine to support routine primary care when there may be a tradeoff, such as between frail older patients staying at home or coming to the hospital for an examination [10,12].

This study reports on the initial experience of a multidisciplinary health care team located at the University Hospital of North Norway (UNN) in using videoconferencing to provide treatment and care to older persons with complex long-term needs during the early months of the pandemic. In Norway, as well as in other countries, there is a rising number of older citizens with complex and long-term health-related needs: “Persons with complex long-term needs typically face multiple care providers, organizations, and specialists, and are especially vulnerable to care fragmentation” [13-16]. This group also dominates the top 5%-10% of spenders, who account for two thirds of high-level health care costs, both in Norway and internationally [17,18]. An extensive body of research indicates that the critical elements of high-quality care for persons with complex long-term needs include strong primary care with an inherent person-centered, integrated, and proactive care focus [19-26]. Despite this, current health care systems are designed for acute short-term needs, and they struggle to address the increasing number of persons with complex long-term needs. Therefore, there is significant interest in the potential of technology such as telemedicine for improving care and reducing costs in these patient populations, as well as in using technology to diminish the burden that individuals face as they manage multiple chronic conditions while adjusting to independent life in the community [10,11,27].

For the multidisciplinary health care team, hereinafter referred to as the Patient-Centered Team (PACT) [19,24], the COVID-19 pandemic has resulted in a reorientation of work practice and a need for a rapid digitalization process to maintain the delivery of person-centered treatment and care to their patients. PACT works as an intermediary team in the transfer phase between the hospital and primary health care and takes a person-centered care approach (see the Research Site section in the Methods for an extensive description of PACT). At the core of person-centered care is the sharing of power between patients and health care professionals, wherein care is driven by the patient’s answer to the question “What matters to you?” The intervention is a continuous process of trust-building, sensitive exploration, and cocreation between professionals and the patient to design and deliver a person-centered care plan.

Accordingly, PACT was concerned about how the use of videoconferencing would affect the person-centered care approach. In the literature, we find that the terms “person-centered care” and “patient-centered care” are used equally; see for instance [19,24-26,28-30]. In this paper, we use “person-centered care” throughout, together with the acronym “PACT” for “Patient-Centered Care Team” as defined in previous papers [19,24].

This case study is based on four semistructured focus group interviews with PACT health care personnel and managers, and we collected data about the experience gained through the period of rapid scaleup of the use of digital care.

The aim of the study is to generate knowledge about the role videoconferencing can play in preserving the person-centered care approach for persons with complex long-term needs. Accordingly, the following research questions have been formulated: How does PACT preserve a person-centered focus on care for persons with complex long-term needs in care services when videoconferencing becomes the main mode of clinical communication due to the social distancing measures during the COVID-19 pandemic? What are the challenges and opportunities for health care personnel in PACT when it is necessary to make a rapid transfer from face-to-face care to video meetings?

## Methods

This qualitative case study was conducted in May and June of 2020. 

### Research Site

To meet the so-called “silver tsunami”—that is, the increased number of persons with complex long-term needs—UNN and Tromsø Municipality, together with neighboring municipalities, established PACT in 2014 at three of UNN’s geographical locations. The teams receive referrals from all health care levels, and they collaborate with health care services, including general practitioners (GPs) who focus on coordinating care in the transition phase between hospitals and municipality care to prevent rehospitalization [19]. It is the aim of PACT to establish a single comprehensive plan for patient follow-up, including hospitals, GPs, and municipal health services. A success factor of PACT is that the teams consist of a combination of hospital and municipality health care personnel (physicians, pharmacists, nurses, secretaries, physiotherapists, and ergonomists) working across organizational borders. They have access to the electronic health record systems in both the hospitals and the municipalities. This provides the team with extensive knowledge and experience to ensure the quality of the transitions; however, it also ensures a comprehensive patient pathway for this group across health care levels and organizational borders. The team takes a person-centered care approach that is aimed at ensuring a holistic understanding in which patients and health care professionals work together toward common goals that are both meaningful to the patient and aligned with what the professionals can offer. To achieve this, the team conducts a risk analysis early in the patient pathway, and the analysis is repeated later in the pathway if there is new information or if challenges occur. In the experience of PACT, the person-centered approach helps to shift the focus away from repairing and treating toward preventing new adverse events or deterioration and initiating early intervention when problems arise [19,24,31]. PACT has achieved excellent results in relation to reducing the risk of death and the number of emergency admissions and hospital stays through the increased use of elective health care services taking the person-centered care approach [19]. The hospital and municipalities share financial, management, and employment responsibilities for the team members [32].

### Participants and Recruitment

This study includes four web-based focus group interviews with a total of 19 participants. The overall project, Dignity Care, had already established collaboration with PACT, and the idea for the paper was first raised in a collaboration meeting, with PACT members expressing concern about how to preserve a person-centered focus when communicating with patients exclusively through digital means. After the study protocol was completed and approved by the Norwegian Centre for E-health Research (NSE), the authors created an information leaflet about the study that included the interview topics. This was sent to the leader of PACT, who forwarded it to all the employees on the team along with an invitation to participate in a focus group interview. The participation in focus group interviews was voluntary, and 19 of the 26 employees in the team (73%) participated. No team member refused to participate; however, some team members were reallocated to other tasks during the COVID-19 pandemic, and others had time off on the day of the interview.

All participants in the study signed an informed consent form and emailed it to the first or second author. The 19 participants were health care personnel or managers of PACT in the age range of 28-62 years, and all but one were female. Many of the participants had over 20 years of experience in health care services, both in hospitals and municipal health care; some had further education in geriatrics psychiatry and interaction, and one participant held a master’s degree in pharmacy. Some of the participants had specific expertise in nutrition for older persons, Parkinson disease, dementia, and management. Further details about the participants in the focus group interviews are presented in Table 1.

**Table 1:** Overview of the focus group interview participants (N=19).

Focus group	Participants, n (%)	Occupations of the participants	Locations	Time of interview
1	7 (37)	Location 1: Hospital nurses (2), municipality nurse Location 2: Physician Location 3: Pharmacist Location 4: Secretary Location 5: Secretary	Tromsø	90 minutes
2	4 (21)	Location 1: Hospital nurse, physical therapist, municipality nurse Location 2: Nurse	Tromsø Balsfjord	60 minutes
3	6 (32)	Location 1: Physical therapist, nurses (3) Location 2: Operations manager Location 3: Medical department advisor	Harstad Senja Narvik	60 minutes
4	2 (10)	Location 1: Leader Location 2: Leader	Tromsø	60 minutes

The focus group interviews were conducted by the first and second authors, who alternated between functioning as moderator and facilitator of the interviews. The interviews were audio-recorded, and reflections were written down immediately after each interview.

We used a semistructured interview guide with three themes and 5-8 subquestions (see Multimedia Appendix 1). The subquestions were used as a checklist to ensure that all the relevant subjects were discussed during the interview. If a subquestion did not come up in conversation, we asked about it specifically. The interview guide was developed in collaboration with all three authors. The themes and subquestions were based on the research questions and on discussions with the third author, who has extensive knowledge from research within the field of patients with complex long-term needs and person-centered care.

The social distancing measures for in-person meetings also affected the design of our study. Accordingly, data were collected through video meetings, as face-to-face fieldwork of any kind was impossible. Focus group interviews conducted on the web are very demanding; some of the participants were in the same meeting room, while others were at other locations, resulting in up to five units being logged on at the same time. There were between 2 and 7 participants in the focus group interviews. Accordingly, the moderator was required to ensure that every participant had time to share his or her experience.

### Analysis

The analysis resembled a systematic text condensation process, a descriptive thematic analysis strategy inspired by Malterud [33]. The four focus group interviews were recorded, transcribed, and then analyzed. First, the authors read the text to establish an overview of the data and identified the overall preliminary themes, including face-to-face practice, rearranging work processes, keeping a person-centered care focus, adapting to technology, and collaboration with other health care professionals. Second, the second author systematically reviewed all the transcribed interviews to identify meaning units and code them in accordance with the preliminary themes. Third, the first and second authors sorted the meaning units according to the preliminary themes. Some meaning units could be sorted into two themes, and these were tagged with a comment to highlight their multiple membership. Fourth, the first and second authors carried out an iterative reading of the systematized meaning units, reducing the content under each theme but maintaining quotations. The first and second authors discussed the condensates and made further adjustments to the text. Based on the previous steps, the preliminary themes were renamed as themes. Citations related to the meaning units were used to ensure that the presented results of the analysis reflected the original context [33]. In addition, in the final phase of writing the article, the first two authors presented the findings of the study to PACT, at which point PACT was able to provide feedback on the results. Prior to submission, all PACT members were offered the opportunity to read the article. We did not receive any objections to what we had presented and written.

### Ethics

For several years, PACT and NSE have been engaged in a research collaboration as a work package in the project Patients and Professionals in Partnership (3P). This study is part of the 3P research project and was approved by the privacy ombudsman at UNN in 2020 (2020/6797). The project was reviewed by the regional ethical committee (REK) (2017/1084/REK nord), which found that the project was exempt from ethical approval.

When the pandemic occurred and digital solutions were being rapidly implemented, PACT requested an evaluation study to assess the consequences for the person-centered care approach when videoconferencing was used for follow-up of their patients. The first and second authors had not previously been involved in the research collaboration and were commissioned to conduct the interviews and data analysis to provide an “outsider” view in the evaluation.

All the participants were provided with a consent form for participation, which they approved in writing and sent to us electronically. Participation in the interviews was voluntary.

## Results

Based on the response in the four focus group interviews, we evaluated how PACT health care personnel and managers experienced the use of videoconferencing to provide person-centered care for persons with complex long-term needs during the initial phase of the COVID-19 pandemic. In accordance with the analysis, the results are presented as condensed text with quotations and are organized according to the following five overall themes: the workflow in PACT before and after the pandemic; technical training and support as prerequisites for effective digital care; new means of collaboration and coordination; divided opinions on using videoconferencing for person-centered patient care; and how saving time on patient travel contributes to person-centered care.

### The Workflow in PACT Before and After the Pandemic

In the traditional way of organizing the work, PACT—as represented by 2-4 health care professionals—would make an initial assessment of the patient’s situation by visiting the patient either while the patient was still in hospital or at the patient’s home. Health care personnel from PACT made 3-4 patient visits per day, in addition to attending collaboration meetings at hospitals or in municipalities. All collaborators, including GPs, homecare services, and health care personnel from the hospital, were required to attend collaborative meetings with the patient and PACT with the aim of coordinating patient services. As a result of the pandemic, however, PACT was required to limit face-to-face meetings with patients and collaborators as much as possible:

COVID-19 was like a sudden wake-up call; we just had to adjust quickly and improvise along the way. We had no choice but to be creative and rethink our work practice.

Participant 7

Normally, the health care personnel in PACT were organized into different teams, although they frequently collaborated with each other and shared their knowledge and experience of treatment and care. Due to the COVID-19 situation, it was necessary to physically separate the PACT team into different cohorts at the hospital, and the physician and pharmacist were required to work from home so that if one team was quarantined, the other team could keep working. There was an overall agreement among the PACT members that this separation hampered collaboration between the teams in terms of the informal sharing of knowledge: “I missed the small talk and information exchange when passing each other in the hallway” (Participant 1).

The team members agreed that the most significant changes to PACT’s workflow when using only videoconferencing for patient communication and care can be summarized as follows: (1) there was less need for ambulation, which saved an extensive amount of time for PACT; (2) the number of health care professionals visiting the patients either at home or in the hospital ward was reduced from 2-4 to 1, with the other necessary team members participating in the meeting by videoconferencing, which saved a lot of resources; (3) there was a reduced need for PACT to visit patients at home, as they collaborated through videoconferencing with assistance from homecare services; and (4) using videoconferencing for collaboration meetings reduced the time spent on travelling to meetings. In addition, collaborators such as GPs were able to attend these meetings more regularly. Finally, PACT found itself performing a support role for hospital ward and homecare services that lacked experience in videoconferencing. These issues are elaborated upon further in the next sections.

### Technical Training and Support Are Prerequisites for Effective Digital Care

Transforming the provision of care from in-person meetings to virtual care demanded extensive training and testing of the equipment, as multiple solutions and setups were being used simultaneously. The goal for PACT was for all members to be able to access and use the different videoconferencing solutions, including Skype, Join, Teams, and Easymeeting, with respect to setting up and running meetings, creating links, choosing the right browsers for the different solutions, and connecting sound and picture. All PACT members had the opportunity to practice and test the different digital solutions during the first month of the COVID-19 pandemic because there were fewer referrals from the municipalities and the hospital. However, although some of the PACT members were comfortable using digital solutions from previous videoconferencing projects, others were not. All team members agreed that the videoconferencing technology itself was the largest barrier to adoption. PACT decided that it was necessary to be comfortable with the technical equipment to integrate videoconferencing into clinical work:

We arranged morning meetings with 8 to 9 different units. At first it took ages before everybody was connected. We were disconnected from the meetings, the sound did not work, the picture froze, you name it… We were given an informal support role, and we used an hour to get everybody online.

Participant 5

All PACT members gained much useful experience from testing and trying out the equipment: “We have learnt that it is smart to test the equipment before a meeting starts to make sure the sound and picture are OK and that the solutions communicate with each other” (Participant 15).

In addition, PACT set out rules for good behavior in videoconferencing, for example, with respect to noise reduction, muting, and taking turns to speak.

The majority of the PACT members had a steep learning curve regarding the use of videoconferencing for clinical purposes, as the technology was challenging. However, the overall perception of the new way of interacting with patients and collaborators was positive: “We all had to think differently about patient care” (Participant 7). This generated innovative initiatives to continue providing person-centered care. When the use of videoconferencing rapidly increased, it led to an extensive need for support for team collaborators as well. PACT reported that health care personnel from other wards and organizations were not as privileged and had less time to familiarize themselves with and test videoconferencing equipment: “In a busy hospital ward, it is unlikely that health care personnel will be allocated time to test technical solutions the way we did” (Participant 9). Moreover, in the hospital, most of the wards had videoconferencing equipment in meeting rooms or offices that could not be used at a patient’s bedside. The PACT members found that when setting up videoconferencing with patients at home, the patients often needed a relative or a health care professional to assist them and provide technical support: “It is important that videoconferencing become available for all patients and not only the ones that are lucky enough to have relatives to assist them” (Participant 14).

Multidisciplinary interaction is key to patient-centered care, and all PACT members stated that they often had to take on a support role to help their collaborators: “In this transitional period, we have been flexible and facilitated the increased use of videoconferencing” (Participant 8). Because videoconferencing was a new way of working for most health care personnel, it was important to make the experience positive for both patients and health care workers: “If the first meeting went well, it was more likely that the patients and collaborators would try videoconferencing for further meetings as well” (Participant 8). PACT had no authority to request that others use videoconferencing; however, they were able to persuade many collaborators and patients to try it, while assisting them along the way. Nevertheless, none of the PACT members considered this support role to be their responsibility in the future use of videoconferencing: “Our focus needs to be on the core competency of patient care. We must be careful not to end up as a videoconferencing helpdesk” (Participant 15).

### New Means of Collaboration and Coordination

#### Collaborating Directly With Patients

There was a general understanding in PACT that introducing videoconferencing for interaction with patients would require close collaboration with homecare services, GPs, and hospital wards. The participants explained that homecare services already had equipment for digital care before the pandemic, namely tablets for documenting treatment and care. During the pandemic, homecare services started using the tablets for videoconferencing meetings in collaboration with PACT. In addition, the physiotherapist from PACT, who was located at the hospital, found that it was possible to assess a patient's mobility and gait function using videoconferencing with assistance from homecare services. Moreover, PACT’s physician and pharmacist, located in their respective home offices, said that they could collaborate with homecare services through videoconferencing to conduct a review of a patient’s medication by “looking into” the medicine cabinets in the patient’s home. Some of the PACT members said that homecare services were also involved in facilitating videoconferencing for the assessment of housing conditions while a patient was still hospitalized, with the aim of preparing the home before the patient’s discharge: “You had the home presented from the patient’s perspective, and they had a more active role in preparing their homecoming while they were still in hospital” (Participant 9).

The participants also said that videoconferencing was used when homecare services needed clinical support from PACT when patients’ conditions acutely worsened. All the PACT units observed that this occurred more frequently than normal during the COVID-19 pandemic. The PACT members assumed that this was related to the patients not being able to attend day care centers and having less contact with GPs and homecare services, whereby changes in patient condition would normally be recognized. During the pandemic, there were times when a patient’s condition would worsen and the homecare services would arrange videoconferencing from the patient’s home with the PACT physician, who would assess the patient. In one case, a patient was admitted to hospital directly, bypassing the normal procedure of first physically visiting a GP. The PACT physician said: “Apart from a clinical examination, I get just as much information about the patient in a videoconferencing meeting as in a face-to-face meeting.”

Videoconferencing was used for several meetings for which a patient normally would have travelled to a hospital. One of the PACT members reported on a stoma follow-up that was conducted at a medical center in a municipality setting through videoconferencing with a stoma nurse at the hospital. PACT members said that some patients also worried about travelling from a municipality with no COVID-19 cases to hospitals in cities with many cases. Over half of the PACT members were satisfied with the patient follow-up on videoconferencing: “We made things happen that we never thought would be possible before we started. It was exciting, it worked very well and people were surprisingly positive” (Participant 10). They also discussed the importance of coming up with good ideas and smart solutions for following up with a patient without physically being in the same place.

Approximately half of the professionals highlighted, from a person-centered care perspective, that using videoconferencing placed the patient in a central position in collaborative meetings involving several actors:

When the patient is at home holding an iPad, the patient is really the one being focused on rather than being just one of many in a face-to-face meeting. In videoconferencing, the patient can easily address everybody directly and thus has a more active and central role. I really liked the idea of having the patient as the center of attention.

Participant 5

Compared with the telephone, most of the PACT members felt they achieved a more personal follow-up using videoconferencing.

#### Coordination Among Health Care Personnel

In the beginning, PACT needed to convince collaborating health care personnel, including both hospital and homecare service personnel and GPs, to start using videoconferencing instead of the telephone: “The health care personnel were just as skeptical, if not even more so, than the patients about using videoconferencing for communication and collaboration” (Participant 3). However, PACT observed that after the collaborating health care personnel used videoconferencing for a while, they felt more comfortable, and it was easier to continue:

We had to work a lot with the wards to get them started. First, they said that their patients were not capable of attending videoconferencing meetings because of their condition and that it was necessary to meet the patients in person, to physically examine them. Nevertheless, they started to rethink when we argued that even physiotherapists made videoconferencing consultations with patients work.

Participant 16

During the first month of the pandemic, synchronous video communication became a valuable tool for interaction between health care personnel in different organizations and geographical locations. The PACT members all agreed that videoconferencing made it easier for collaborators such as GPs, homecare services, and service administrators (*forvaltingskontor*/*tildelingskontor* in Norwegian) to attend on a regular basis because they saved time on transportation and parking: “We have been even more multi-disciplinary than before the pandemic at coordination meetings” (Participant 14). There was general agreement in PACT that GPs in particular attended videoconferencing coordination meetings more regularly than they did when physical meetings were the only option. In addition, some PACT members said that it was easier to arrange meetings at short notice when participants could attend from their offices. There was general agreement in PACT that collaborative meetings on video were shorter than in-person meetings. Some of the PACT members elaborated, saying that collaborating health care personnel seemed to be better prepared for videoconferencing meetings and more focused on the problem to be addressed, and there was less small talk: “I think video meetings lasted only half as long as in-person ones; they were both time- and cost-saving” (Participant 19). Despite this, all PACT members found that videoconferencing was more demanding and intense than physical meetings, and some of them recommended limiting the meetings to 1 hour.

All PACT members agreed that introducing videoconferencing for collaboration across organizations improved the interorganizational relationship between all the actors involved in treating a patient. PACT had extensive collaboration with homecare services, as mentioned previously, as homecare services were the only professionals still making regular face-to-face patient visits. However, several PACT members reported that the personnel in hospital wards often seemed to find the technology challenging and preferred for PACT to configure the tablet used for videoconferencing at the patient’s bedside. They further stated that health care personnel often assisted and supported patients in the wards in digital meetings. This was highly successful, as the ward professionals could add supplemental information to the patient's story and any ambiguities could be clarified. All PACT members emphasized that the hospital wards should have their own tablets for reasons of infection control, and that in the long run, the staff should have proper training so that they could manage videoconferencing equipment themselves. Although all PACT members were satisfied with being able to reach out to their patients using videoconferencing, there were pros and cons regarding its extensive use.

### Different Opinions on Using Videoconferencing for Person-Centered Patient Care

The PACT members had different opinions on how well the person-centered focus was preserved when videoconferencing replaced face-to-face follow-up. Some found it difficult to work digitally when assessing patients. For example, if a patient was confined to bed in the hospital, it was difficult to obtain an overall impression of the patient's physical status through a screen:

I think physical meetings present a much broader view of the patient’s status. My impression is that many patients “straighten up” for a few minutes during a digital visit. When you are in the same room, it is easier to assess the patient and evaluate his condition.

Participant 2

On the other hand, another team member said, “We had an extensive number of videoconferencing meetings over the last few months, and I think they worked extremely well, both for collaboration and for patient meetings” (Participant 9). PACT ran several multidisciplinary meetings to follow up with patients: “It was exciting and worked very well, and people were surprisingly positive” (Participant 11).

Persons with complex long-term needs are a heterogeneous group, and there was general agreement in PACT that videoconferencing was not a good solution for all of them. The health care personnel in PACT had a range of opinions on the potential of using videoconferencing to preserve person-centered care for this patient group. Some of the PACT members found it demanding to communicate by video with patients who had vision or hearing impairment. They reported that patients with hearing impairment had problems with verbal communication using a tablet. Other PACT members recognized that digital meetings could be challenging for patients with reduced cognitive capacities. One patient, for example, had difficulty understanding that the health care personnel participating by videoconferencing were actually “live” in the same room and not just a picture on the wall: “Oh yes, I remember her, when are we going to see her?” (Participant 1). All PACT members emphasized that patients with cognitive impairments often needed health care personnel or relatives to support them in videoconferencing. Hence, PACT reported that if health care personnel did not find that patients were competent to participate in video meetings, the patients were sometimes excluded from the meetings. However, other PACT members reported the opposite, namely that videoconferencing made it possible to include even the frailest patients from home or from the hospital:

We, the health care personnel, often have the most prejudice about which patients to include in videoconferencing. However, we had meetings with several patients with cognitive impairments and other disabilities that worked surprisingly well.

Participant 7

Another complicating issue brought up by some of the PACT members was the difficulty of preserving privacy in videoconferencing meetings with bedridden patients admitted to multibed patient rooms in wards: “It is a challenge that hospitals lack rooms with videoconferencing that are adapted for patients confined to bed” (Participant 15). However, there was common agreement among the PACT members that videoconferencing was a good way of keeping in contact with patients and maintaining collaboration amongst health care personnel in the demanding situation of the COVID-19 pandemic: “It is much more complex to work digitally; still, it is better than not reaching patients at all” (Participant 3).

### Saving Time on Patient Travel Contributes to Person-Centered Care

All PACT members emphasized that providing person-centered care for persons with complex long-term needs is also about limiting the health care disruption to patients’ daily lives as much as possible. PACT highlighted the importance of seeing the patient as an individual person and not just as an accumulation of different diseases: “For us health care workers, the disease often defines the patient. For the patient, the disease is just a part of their life, not all of it” (Participant 15). All PACT members conducted their work from a person-centered care focus, in terms of starting the collaboration by asking the patient “What matters to you?” and translating the answers into health-related goals.

Some PACT members mentioned that efficiency and cost savings have been important focus areas in recent years; however, saving patients’ time should be just as valuable. They added that several persons with complex long-term needs struggle with mobility challenges with their diseases; accordingly, travelling takes much time and energy. PACT reported that many appointments and meeting cancellations might have been avoided through the use of virtual communication: “If the patient is allowed to exchange the long, exhausting trip to the hospital with a 15-minute videoconferencing check-up, this is an important person-centered gain” (Participant 15). PACT elaborated that for some patients, it takes days to recover from travelling all day: “We had so many positive results from using videoconferencing that we believe many patients can reduce the number of trips they make to the hospital for follow-up” (Participant 9).

## Discussion

### Principal Findings

In the Results section, we present the empirical findings from the focus group interviews on how PACT experienced the change in their work practice from using videoconferencing for interaction with persons with complex long-term needs and with their relatives and health care collaborators when providing person-centered care. In summary, some PACT members found it very challenging to use videoconferencing for patient communication and wanted to return to the traditional way of working. Others thought that videoconferencing worked very well for all patient groups and found it inspiring to use videoconferencing as a means of continuing a person-centered approach for treatment and care under challenging distancing measures. The largest challenge with using video meetings was the technology itself and the unpredictability of making the technology work. PACT emphasized the need for extensive training and support, as well as for just one video solution for collaboration between different organizations. The greatest advantages of videoconferencing were the new opportunities for collaboration between patients and PACT, the related significant time saving on travelling for both parties and the better use made of the limited resources of health care personnel. The improved collaboration with other health care services was also highlighted. On the basis of the empirical findings presented, we will discuss the role of videoconferencing in preserving a person-centered care approach related first, to improving personalized pathways; second, to reorganizing work practice; and third, to addressing new support roles. As a frame for the discussion, we used the previous research presented in the Introduction and the experience of the three authors (health care personnel and researchers).

### Video Meetings Improve the Personalized Patient Pathway

Health care organizations are increasingly shifting from a disease-centered to a person-centered care focus. A person-centered care approach is believed to enhance both the technical and patient-experienced quality of care and to better achieve the quadruple aim of improved care experience, health and function, cost-benefit ratios, and improved work life for those who deliver care [34]. There is no agreed-upon definition or method of measurement for person-centered care [28]. It is an approach which includes quality dimensions best assessed by the patient. However, this approach addresses how a fragmented care system can create a seamless, personalized pathway that addresses a person’s needs, values, and preferences as they develop over time [24]. The results show that PACT found that videoconferencing worked well as a means of improving collaboration and communication in person-centered care: “For most patients, the person-centered care follow-up has been at least as good as normal when using videoconferencing” (Participant 7).

Efficiency and cost savings have been important focus areas in recent years [11,27]; however, saving the patient’s time should be just as valuable. Previous research demonstrates that telemedicine can be used to replace referrals to an outpatient clinic, thus reducing travel and unnecessary hospital visits, especially for those living in remote areas [10,11]. Using video meetings eliminates the risk of costly cancellations, such as those due to bad weather such as snowstorms leading to closed roads and cancelled flights; this is a constant risk in northern Norway, where the winter is approximately 6 months long. The patient can avoid unnecessary travel time [2], which is beneficial for this fragile patient group. Technological solutions such as videoconferencing can support persons with complex long-term needs by allowing improved access to different parts of the fragmented care system, which can be adjusted to the person’s needs and preferences as these develop over time [11,19,24,27]. A number of hospital consultations and follow-ups can be changed to video meetings. Hence, several PACT members stated that videoconferencing can help facilitate living with complex disorders and reduce the disruption of the patient’s everyday life due to the disease as much as possible, which we believe is an important principle in further developing the concept of person-centered health care and which is in line with Bower et al [27,35].

As elaborated upon in the results section, persons with complex long-term needs are a heterogeneous group, and the use of videoconferencing was challenging for patients with vision and hearing impairments as well as for those with cognitive impairments. Greenhalgh et al [10] state that not all clinical situations are appropriate for video consultations. This is in line with the feedback from PACT and raises the need for guidelines on how to tailor the use of videoconferencing to different patient capabilities and disabilities [10].

### Video Meetings Require Reorganization of Work Practices

Digital care can improve the use of fragmented care system resources [11,19,27]. PACT includes a combination of disciplines, such as physicians, nurses, and physiotherapists. When PACT visits patients at home, normally, they require 3-4 people to be present. During the pandemic, they reduced the number of team members visiting patients at first to 0 and then to 1 or 2. Using videoconferencing as a supplement reduced the need for outreach resources, as they could contact additional team members virtually when needed. They found this approach to be a more efficient and resource-saving way of organizing their multidisciplinary work. In addition, it was easier for other collaborating health care personnel in the municipality, hospital, and GP offices to participate in collaboration meetings by using videoconferencing from their offices, as this saved them from spending time traveling to other locations. Furthermore, as PACT members have stated, videoconferencing meetings were more “to the point” in terms of focusing on patient needs, all participants came prepared, and meetings were completed within the allocated time: “We did much more than expected and saved a great deal of time, even though we were not physically in the same place” (Participant 8).

Patient pathways for persons with complex long-term needs include all levels of health care services. Therefore, using videoconferencing to improve the fragmented care system requires that all actors be engaged, for which reason using the potential of videoconferencing requires an overall rearrangement of health care services so that videoconferencing is included as part of daily services [11,27]. This is in line with previous studies (eg, Alami et al [3]), which found that using digital care calls for organizational changes in processes, practices, cultures, communication, and the division of work. As stated in the introduction, previous research has identified three major themes that affect the implementation and use of telemedicine. The results from this study also support the importance of these themes when scaling its implementation in an organization. Using the three themes to understand the empirical results of this study is helpful for focusing on the premises for using videoconferencing, which are to not only preserve but also improve the person-centered approach for persons with complex long-term needs. The three themes are discussed below.

#### Theme 1: Governance and Strategy

The fragmented portfolio of videoconferencing solutions made it challenging for health care personnel to connect to meetings. All the PACT members stated they would like health care organizations to standardize the process to use only one video-meeting solution, as compared to the many solutions currently used. The PACT members said that the complex technology created a feeling of incompetence and of being unprofessional as health care personnel, which created a barrier to using videoconferencing.

#### Theme 2: Organizational and Professional Dimensions

Conducting videoconferencing meetings led to additional work for PACT. For example, PACT members were required to set up tablet computers for hospital wards and take on an information technology support role for other health care units. Moreover, in this empirical case, PACT were the ones coordinating the collaboration around the patients; hence, they continued this coordination in virtual meetings. Nevertheless, it is important to discuss and clarify who has *legal* responsibility when assessments, treatment, and care decided upon in virtual meetings must be conducted on behalf of an actor from a different organization (eg, when homecare services monitor blood pressure or an electrocardiogram on behalf of specialist health care).

#### Theme 3: Economic and Financial Dimensions

Videoconferencing shows the need for clarification of the regulations for reimbursement. In Norway, during the early phase of the COVID-19 pandemic, the reimbursement for videoconferencing was changed so that it was equal to that for face-to-face meetings in view of the increasing use of digital care. This covered the use of videoconferencing for outpatient consultations and appointments with GPs. Nevertheless, as described above, when actors from one organization conducted assessments and procedures on behalf of an actor from another organization, this could raise the question of reimbursement for the executing party. In addition, the question arose of who was responsible for financing the technical support when actors from different organizations collaborated on caring for the same patient. These issues were barely mentioned by PACT; however, in line with previous research, they are still important to highlight [3]. In addition, the potential of telemedicine in general to improve care and reduce costs is limited by a lack of rigorous evidence of actual impact [27].

### Scaling Up Digital Care Creates a Need for New Support Roles

The results of this study show that new roles that support the technical use of telemedicine for health care personnel must be established as part of using videoconferencing to improve a person-centered care approach. This technical support must work in close collaboration with personnel at all organizational levels of health care:

It is important to organize high-quality support. If you have five doctors in the meeting and it takes 15 minutes to connect everyone, you have wasted more than an hour in total. You don’t have to spend many of these 15-minute periods before you have spent the equivalent of paying a technician to handle the practical and technical issues related to videoconferencing.

Participant 6

The support must be readily available in terms of a short response time: “When you have video meetings, there is a risk that you will focus more on the technology and how to make everything work than on patient issues and the other actors in the meeting” (Participant 16). PACT was concerned that it would be locked into a future support role and would be in danger of compromising its focus on patients. PACT cannot maintain this support role; however, the support organization must have the clinical competence to understand clinician needs.

As described in the results, patients often needed assistance from homecare services or relatives to connect to videoconferencing. Not all patients are familiar with synchronous video communication, and not everyone has relatives to support them. One suggested solution was to extend the collaboration with homecare services to facilitate and support such meetings. However, this would create new work tasks for health care personnel in a care organization, which raises the aforementioned question of reimbursement and training of homecare personnel. Accordingly, scaling up the use of digital care has wide-ranging implications of a clinical, organizational, and economic character [11].

In sum, the COVID-19 crisis has shown that the health care service needs an alternative approach to face-to-face treatment and care. Based on the clinical perspectives in this study, the use of videoconferencing requires a long process of learning and adaptation, both individually and collectively, to ensure its successful integration into an organization [11]. The use of telemedicine must be recognized as an organizational development issue [3], in which the scaling up of videoconferencing creates challenges at different stages of its deployment [8].

### Future Research

For the continued integration and sustainable use of videoconferencing to improve person-centered care, further research must be conducted that takes into consideration the complexities of individual patients, the need for tailored technical support, organizational factors, and economic issues. The first step is to interview PACT about its use of videoconferencing for more than the half-year following its rapid implementation and to investigate the use of videoconferencing as the COVID-19 pandemic subsides in this part of the world. It is also important to interview patients and PACT collaborators, such as homecare services and GPs, to evaluate their experience.

### Limitations

In this study, we interviewed a specific group of health care personnel, namely PACT. PACT’s experience of using digital care relied extensively on the adoption of videoconferencing by both its collaborators and patients. Although different health care professions are represented in PACT, a more generic view of the experience of preserving a person-centered care approach during the rapid digitalization and use of videoconferencing would have been gained from its collaborating partners in other organizations, such as homecare services, and from GPs and specialists in hospitals. In addition, we did not interview any patients; therefore, all the patient-related responses are secondhand knowledge as reported by PACT. To describe the person-centered care focus, we would need to interview patients directly as well.

### Conclusions

Scaling up the use of digital care has been a goal in Norwegian health care for years. The COVID-19 pandemic and the rapid adoption of digital care have created a unique opportunity to continue developing a health service for not only preserving but also improving the person-centered care approach for persons with complex long-term needs.

Using digital care has provided an opportunity for more time-saving and efficient follow-up and coordination of this patient group in terms of reduced traveling time for patients and the more efficient use of health care personnel in PACT, as well as for improving the information exchange between health care levels.

In this study, we found that digital care cannot replace face-to-face interaction between patients and health care personnel. All the PACT members underscored the importance of face-to-face patient interaction as the foundation of care and that videoconferencing was a supplement to enhance follow-up and coordination. However, scaling up the use of digital care requires overall agreement and collaboration among the different health care levels and actors in terms of training, guidelines for when to use digital care, procedures for its use, technical support, etc, to ensure the success of digital health care services. Videoconferencing can then contribute to better preparing the health care services for future scenarios, such as new waves of COVID-19 or similar outbreaks, where there may be a need for social distancing in public and health care services.

## Appendix

Multimedia Appendix 1 Interview guide.

## Abbreviations

3P: Patients and Professionals in Partnership
GP: general practitioner
NSE: Norwegian Centre for E-health Research
PACT: Patient-Centered Team
UNN: University Hospital of North Norway
