# Supplementary material for: Preservation of Person-Centered Care Through Videoconferencing for Patient Follow-up During the COVID-19 Pandemic: Case Study of a Multidisciplinary Care Team
Source: JMIR Form Res. 2021 Mar 5;5(3):e25220. doi: 10.2196/25220 (PMC7939056; doi:10.2196/25220)
Supplement: Multimedia Appendix 1 [file formative_v5i3e25220_app1.doc]

Interview guide

**From a Healthcare Perspective: How to Preserve Person-Centred Care Using Video Conference to Follow Up with Patients during the Covid-19 Pandemic**

## Research question:

How does PACT preserve a person-centred care focus for persons with CLN in care services, when VC becomes the main mode of clinical communication, due to social distancing measures during the Covid-19 pandemic?

What are the challenges and possibilities for healthcare personnel in PACT when a rapid transfer from face-to-face care to video meetings is needed?

## Theme 1: Health professionals’ experiences and challenges with an abrupt transition to digital collaboration.

- Can you describe the person-centred care approach to CLN patients before and during the Corona pandemic, what can be done using VC and what is more challenging to conduct using VC?
- Is there a difference between working with patients that is known by you (PACT) from before the pandemic versus new patients referred during this period?
- Are you comfortable with the digital format (VC) yourself; is the technology difficult, do you have the necessary equipment available?
- Do you get the same benefits from patients assessments/meetings when using VK?
- Which experiences can be useful to include in further digitalization processes?
- How has increased digitalization changed your work processes?
- How do you follow up the patients who still needs physical measures when personal contact from homecare services and other healthcare organizations is reduced?
- During increased digitalization of healthcare services, how is the person-centred care focus maintained in a secure, comprehensive and proactive health and care service?

## Theme 2: Healthcare professionals experience of how patients/relatives handle digital interaction

- What feedback did you get from patients/relatives after increased use of VC/digital care?
- Which patients handled the transition to VC/digital care tools best/worst?
- Do you get feedback from patients/relatives on how VC works?
- What do you do if patients do not want/are comfortable with using VK?
- Have patients gained increased access to medical technical equipment for home monitoring, for example, blood pressure monitors?

## Theme 3: Have the increased digitalization influenced the way healthcare services are organised and the way they collaborate?

## Have the digitalization and use of VC influenced the collaboration in one way or another?

## E.g. have VC made it easier or more cumbersome to gather actors from different health services that are collaborating on a patient meet for planning treatment, follow-ups and care?

## Is it possible to use multi-party VCs that enable several collaborators from different organizations to participate in the same meeting together with a patient?

- Are the cooperating organizations prepared for increased use of digital interaction? Why/ why not?
- Do you think that digitalization will continue after the Corona pandemic stabilize?
- How do you think an increased use of VK and digital tools will change the way you collaborate and work together with persons with complex, long-term needs over time?
- Are there any differences in how VC is used in different parts of the healthcare service (hospitals, home care, nursing homes, GPs etc)? E.g. do homecare services have digital tools such as Ipads to bring out to their patients?
